# *Wedelia trilobata* (L.) Leaf Extract Induces Autophagy-Mediated Cell Death in HT-29 Colorectal Cancer Cells via Suppression of the Akt/mTOR Signaling Axis

**DOI:** 10.3390/ijms27104636

**Published:** 2026-05-21

**Authors:** Tue Minh Duong, Thanh Chau Quoc Nguyen, Tomonori Waku, Kenji Kanaori, Kaeko Kamei

**Affiliations:** 1 Department of Functional Chemistry, Kyoto Institute of Technology, Kyoto 606-8585, Japan; 2 Faculty of Chemistry, College of Natural Sciences, Can Tho University Campus II, 3/2 Street, Ninh Kieu Ward, Can Tho City 94000, Vietnam

**Keywords:** *Wedelia trilobata*, anti-colorectal cancer, autophagy, Akt pathways

## Abstract

Colorectal cancer remains a global health challenge due to its high mortality and therapy resistance. While *Wedelia trilobata* (L.) (WT) exhibits pharmacological potential, its specific mechanisms against this cancer are not fully understood. We investigated the anticancer effects of *W. trilobata* leaf ethanol extract and its *n*-hexane and chloroform fractions on HT-29 cells. The WT extract significantly inhibited proliferation by inducing G1/S phase arrest and downregulating *PCNA* mRNA. It triggered substantial DNA damage (increased γ-H2AX) and suppressed the mitogen-activated protein kinase (ERK) pathway. Notably, the WT extract-induced autophagy-mediated cell death, marked by acidic vesicular organelle formation and increased LC3-II levels. Inhibition of autophagy with *N*-acetylcysteine and 3-methyladenine partially rescued cell viability, restored p-Akt levels, and reduced LC3-II, indicating that cell death is regulated via the ROS-mediated Akt/mTOR signaling axis. Additionally, autophagic flux was validated using chloroquine, which led to a synergistic accumulation of LC3-II. GC-MS analysis identified 48 and 52 compounds in the *n*-hexane and chloroform fractions, respectively, including metabolites with known antioxidant and antitumoral properties. These findings demonstrate that *W. trilobata* induces autophagic cell death through ROS-mediated Akt/mTOR inhibition, supporting its potential as a source of innovative colorectal cancer therapeutics.

## 1. Introduction

Colorectal cancer (CRC) is the third-most diagnosed cancer and the second-most common cause of cancer death worldwide [1]. By the year 2030, an estimated 2.2 million patients will be diagnosed with colorectal cancer, and the global burden of this disease is expected to increase by 60% [2]. High rates of metastasis and invasion render colorectal cancer a particularly difficult malignancy to treat effectively. Although the five-year survival rate remains high (90%) for localized tumors, this figure decreases drastically to approximately 14% for patients with distant organ involvement [1]. The clinical burden is further highlighted by the fact that 25% of cases are metastatic upon diagnosis, with 50% of all colorectal cancer patients ultimately developing distant lesions during the course of disease [3]. Unfortunately, despite advancements in surgical techniques, radiotherapy, and chemotherapy, the effective treatment, prevention, and combat against metastatic spread remain severe challenges. Indeed, most patients with colorectal cancer eventually die of metastatic progression [4]. Researchers are therefore exploring novel therapeutic agents and diverse strategies for the treatment and prevention of colorectal cancer and various other malignancies.

Recent insights into the role of p53 in CRC underscore that mutations in the *TP53* gene occur in over 50% of CRC cases and are a primary driver of chemoresistance [5]. The HT-29 cell line, a *p53*-mutant colorectal adenocarcinoma model used in this study, is characterized by its high resistance to radiation and its clinical relevance in drug discovery. Given its *p53*-deficient status, HT-29 serves as a critical model for identifying novel bioactive compounds capable of bypassing classical apoptotic resistance.

The mitogen-activated protein kinase (MAPK) network orchestrates key events in colorectal cancer development. This system is subdivided into the ERK, JNK, and p38 signaling modules, each serving unique functional roles [6]. ERK activation typically underpins cell proliferation and cytoskeletal reorganization, while the JNK pathway integrates signals to determine cell fate through proliferation or death [6]. Furthermore, the p38 kinase is central to the regulation of metastasis, cell cycle checkpoints, and cellular differentiation [7]. In addition, the Akt/mTOR signaling pathway plays a crucial role in the growth, proliferation, angiogenesis, invasion, and migration of tumor cells. These pathways are known to be key targets for molecular-targeted therapy.

A growing body of evidence suggests that natural products and herbal extracts offer unique advantages and minimal side effects, either by improving the cancer microenvironment or enhancing the anticancer effect in treating cancer. *Wedelia trilobata* (L.) (also known as *Sphagneticola trilobata* (L.)) is a member of the family Asteraceae known for its therapeutic effects on ulcers, sore throats, varicose veins, headaches, fevers and indigestion. It is a fast-growing, prostrate perennial herb that forms dense mats and has become naturalized in diverse tropical regions. Some literature has reported the plant’s bioactivities, including antioxidants, anti-inflammatory [8], antibacterial [9], and antifungal properties. Further, Mardina, V et al. demonstrated the anticancer activity of its methanol and *n*-hexane extracts on breast cancer [10,11]. Previous studies by Fagundes, T.R. et al. have identified the anticancer potential of grandiflorenic acid, a major constituent of *W. trilobata* (L.) leaf extract, across various cancer models [12].

However, the specific inhibitory mechanisms of *W. trilobata* regarding colorectal cancer proliferation and metastasis remain poorly understood. While Chi H.T. et al. recently reported that the methanol extract of this species induces apoptotic cell death via the suppression of BCR/ABL and subsequent modulation of Akt and MAPK pathways in human leukemia cells [13], its effects on solid tumors remain to be characterized. Based on these findings, we hypothesized that *W. trilobata* leaf extract might target similar survival signaling axes in colorectal cancer cells.

The present study demonstrates that *W. trilobata* extract significantly suppresses the migration and proliferation of HT-29 human colorectal cancer cells. Through solvent partitioning, we identified that both *n*-hexane and chloroform fractions possess potent anticancer activities. Notably, phytochemical analysis revealed that grandiflorenic acid was present in the *n*-hexane fraction but absent in the chloroform fraction. Furthermore, we demonstrated a cell death mechanism that differs from previous reports, specifically focusing on autophagy-mediated pathways.

## 2. Results

### 2.1. WT Leaf Extract Decreases Viability of HT-29 Cells

**Figure 1:**
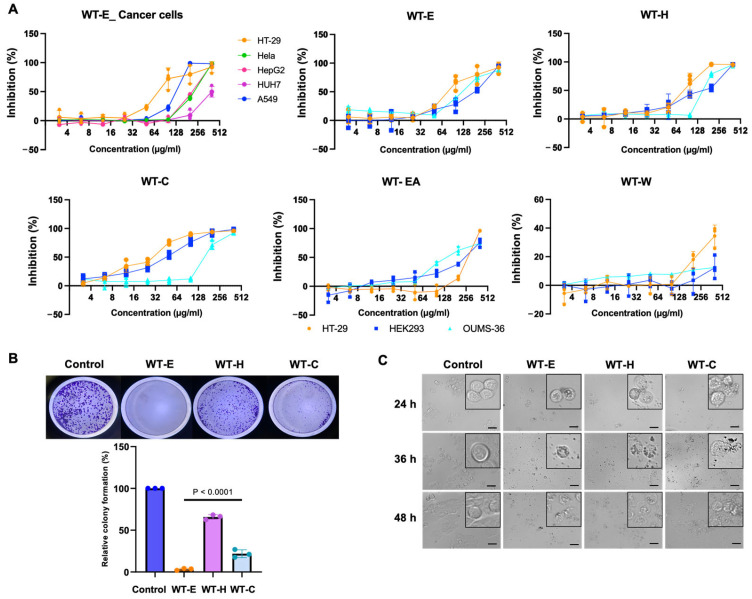
Effects of WT extract and its solvent fractions on cell viability of cancer cells and normal cells. (**A**) Cancer cells, various kinds of cancer cells (HT-29, HeLa, HepG2, HUH7, A549) were seeded at 5 × 10^4^ cells/well in 96-well plates and treated with various concentrations of WT-E for 48 h. HT-29 cells (5 × 10^4^ cells/well), HEK293 cells (10^5^ cells/well) and OUMS-36 cells (10^5^ cells/well) were treated with various concentrations of WT-E or each solvent fraction for 48 h. Cell viability was measured using a CCK-8 assay kit. The IC_50_ values were determined by nonlinear regression analysis with GraphPad Prism software. (**B**) Clonogenic potential of HT-29 cells. Colonies were counted after treatment with WT-E. WT-H and WT-C at respective IC_50_ value concentration or without additives (control) for 10 days. Data are represented as mean percentage of viable cells in bars ± S.D. (*n* = 3). (**C**) Morphological changes in cells treated with WT samples at their IC_50_ concentrations for 24, 36, and 48 h. Images were captured using phase-contrast microscopy. Scale bar = 50 μm.

To evaluate the anticancer potential of the *Wedelia trilobata* (L.) leaf ethanol extract (WT-E), cell viability was assessed across a panel of human cancer cell lines, including HeLa (cervical), HepG2 and HUH7 (hepatocellular), A549 (lung), and HT-29 (colorectal) adenocarcinomas. HEK293 (human embryonic kidney) and OUMS-36 (normal human embryonic fibroblasts, a well-established model for assessing general cytotoxicity in human cell lines) were utilized as non-transformed control lines. Cells were treated with varying concentrations of WT-E (3.13 to 400 μg/mL) for 48 h, and cytotoxicity was determined using CCK-8 assays. As illustrated in Figure 1A, WT-E reduced cell viability in a concentration-dependent manner. Using nonlinear regression analysis, the IC_50_ values were calculated as follows: HT-29 exhibited the highest sensitivity to WT-E (IC_50_ = 84.38 μg/mL), followed by A549 (123.52 μg/mL), HepG2 (222.35 μg/mL), and HeLa (211.77 μg/mL), while HUH7 cells showed minimal sensitivity (IC_50_ > 400 μg/mL). Regarding the normal cell lines, the IC_50_ values were 157.79 μg/mL for HEK293 and 148.10 μg/mL for OUMS-36. The Selectivity Index (S. I.) was calculated to evaluate the preferential toxicity of WT-E. The S. I. values for HT-29 cells were 1.87 (relative to HEK293) and 1.76 (relative to OUMS-36). Based on its superior sensitivity and favorable selectivity profile, the HT-29 cell line was selected for further mechanistic investigations.

To identify the most bioactive components, WT-E was subjected to solvent partitioning, yielding four distinct fractions: *n*-hexane (WT-H), chloroform (WT-C), ethyl acetate (WT-EA), and water (WT-W). The cytotoxicity of each fraction was subsequently evaluated against HT-29 cells. After 48 h of treatment, WT-H and WT-C exhibited potent antitumor activity, with IC_50_ values of 79.72 μg/mL and 24.53 μg/mL against HT-29 cells, respectively (Figure 1A).

Regarding the non-transformed cell lines, WT-H showed IC_50_ values of 133.35 μg/mL (HEK293) and 166.90 μg/mL (OUMS-36). Notably, WT-C demonstrated a superior safety profile, with IC_50_ values of 47.24 μg/mL (HEK293) and 173.70 μg/mL (OUMS-36). Consequently, the S. I. values for WT-C were calculated at 1.93 for HEK293 and an impressive 7.08 for OUMS-36, both of which were significantly higher than those of WT-E (1.87 and 1.76, respectively) and WT-H (1.67 and 2.09, respectively). In contrast, the WT-EA and WT-W fractions exhibited IC_50_ values exceeding 300 μg/mL against HT-29 cells, indicating relatively low cytotoxicity. Based on these findings, WT-E, WT-H, and WT-C were selected for subsequent mechanistic studies at their respective IC_50_ concentrations.

The antiproliferative activity of the extracts was further evaluated using the clonogenic survival assay. The colony-forming ability of the HT-29 cells was remarkably reduced after 10 days of treatment at the IC_50_ concentrations of WT-E, WT-H and WT-C (Figure 1B). The effects of the WT samples on the morphology of HT-29 cells were observed using phase-contrast microscopy. As shown in Figure 1C, during the initial 24 h treatment with the WT samples, the morphology of the HT-29 cells was indistinguishable between the treated and non-treated groups. In contrast, the morphological changes in the HT-29 cells were observed after 36 and 48 h treatment with the WT samples, resulting in cell shrinkage and an increase in the suspended cell population.

**Figure 2:**
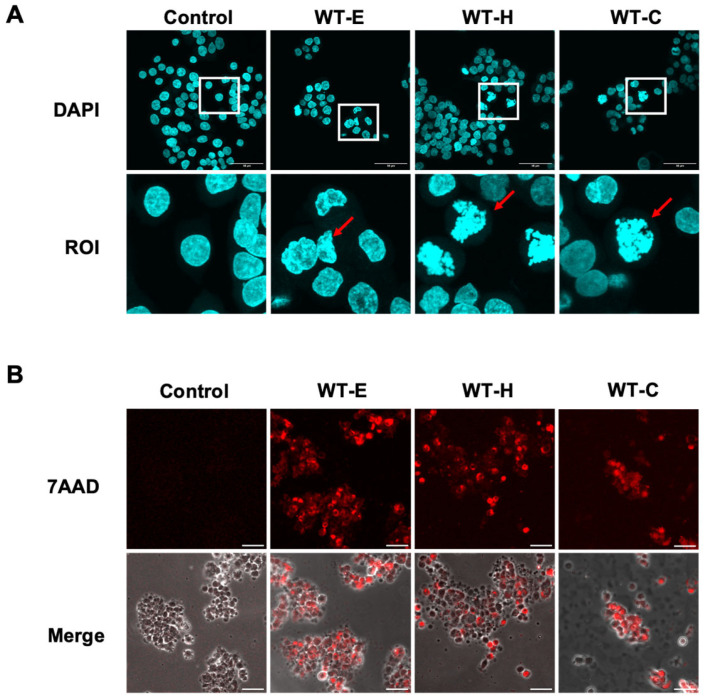
Effects of WT leaf extract on HT-29 cell morphology and viability. HT-29 cells were treated for 36 h with WT-E, WT-H, and WT-C at their respective IC_50_ concentrations, followed by washing and staining. Cells were then observed under a fluorescence microscope. (**A**) DAPI staining of HT-29 cells. White squares indicate magnified regions of interest (ROIs). Red arrows denote nuclear condensation and fragmentation. (**B**) 7-AAD staining of HT-29 cells. Fluorescence images were merged with differential interference contrast (DIC) images. Scale bar = 50 μm.

DAPI staining revealed distinct nuclear condensation and fragmentation in HT-29 cells after 48 h of treatment with WT samples, indicating nuclear damage (Figure 2A). In addition, 7-AAD staining showed increased intensity of red fluorescence in WT-treated cells compared with the control group, confirming loss of membrane integrity and cell death (Figure 2B). These findings suggest that the WT extracts induce nuclear alterations and promote cell death in HT-29 cells.

### 2.2. WT Leaf Extract Suppresses Migration of HT-29 Cancer Cells

**Figure 3:**
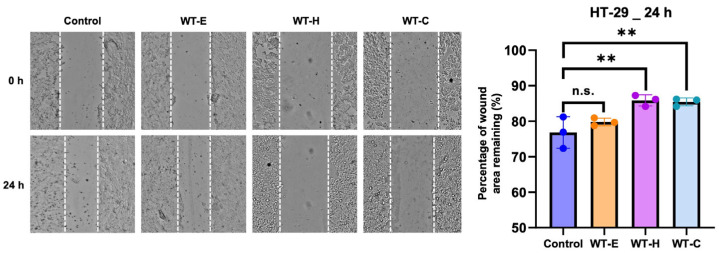
Inhibition of HT-29 cell migration by WT leaf fractions. The cells were treated for 24 h with the respective IC_50_ value concentrations of WT-E, WT-H and WT-C. The white dotted lines delineate the advancing boundaries of the migrating cell sheets used to quantify wound closure. Relative migration distances were measured three times for each scratch. **, *p* < 0.01 when compared with the control group; n.s., not significant. The data are presented as the mean ± S.D. (*n* = 3).

To evaluate the effects of WT extract on the migration of HT-29 cells, a wound healing assay was performed. Compared to untreated cells, the reduction in gap width was significantly suppressed following 24 h of treatment with WT-E and its solvent fractions, WT-H and WT-C, at their respective IC_50_ concentrations (Figure 3). Control cells occupied approximately 25% of the wound area after 24 h. In contrast, the percentage of wound closure was significantly reduced in the groups treated with WT-H and WT-C, whereas WT-E exhibited no significant effect. These results suggest that WT leaf extract-derived fractions may inhibit the migratory capacity of HT-29 cells, potentially contributing to the suppression of cancer metastasis.

### 2.3. WT Leaf Extract Induces Cell Cycle (G1/S) Arrest of HT-29 Cells

**Figure 4:**
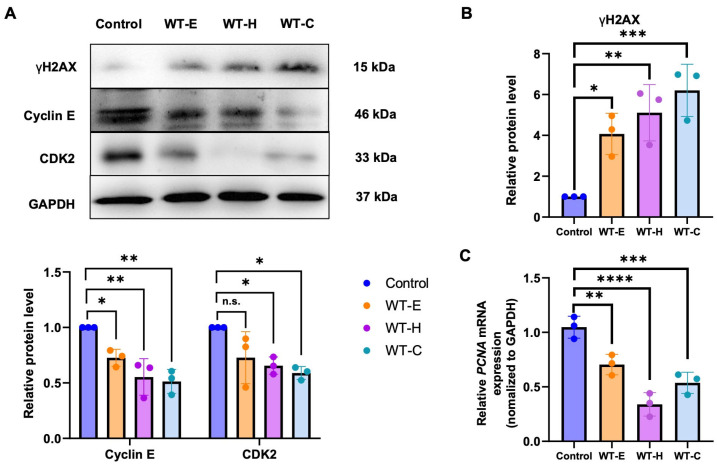
Induction of G1/S phase arrest in HT-29 cells by WT leaf extracts. HT-29 cells were treated with WT-E and its solvent fractions at their respective IC_50_ concentrations for 36 h. (**A**) Western blot analysis of Cyclin E, CDK2 and γH2AX (Phospho-Ser139) and densitometric quantification of Cyclin E and CDK2 band intensities. (**B**) Densitometric quantification of γH2AX (Phospho-Ser139) band intensities using ImageJ software (version 1.54j). (**C**) RT-qPCR analysis of *PCNA* mRNA expression levels. Relative protein and mRNA levels were normalized to the control group (100%). GAPDH was used as the loading control for both assays. Data are presented as the mean ± S.D. (*n* = 3). Statistical significance: * *p* < 0.05, ** *p* < 0.01, *** *p* < 0.001, and **** *p* < 0.0001 vs. control; n.s., not significant.

DNA double-strand breaks (DSBs) are well-characterized critical lesions that compromise genomic stability, frequently stemming primarily from DNA damage during replication. To investigate this, H2AX phosphorylation (γH2AX)—a robust marker for DSBs—was analyzed by Western blotting in HT-29 cells following 24 h of treatment with WT extract at IC_50_ concentrations. The results demonstrated that the levels of γH2AX (Phospho-Ser139) were significantly increased upon treatment with WT solvent fractions (Figure 4B).

Furthermore, compared to the untreated control, groups treated with WT extract and its solvent fractions showed reduced transcription of the *PCNA* gene, which is essential for DNA replication and cell cycle progression (Figure 4C). Since the Cyclin E/CDK2 complex plays a vital role in the G1-to-S-phase transition, we examined its protein expression levels to explore the molecular mechanisms underlying the observed arrest. Western blot analysis revealed that both Cyclin E and CDK2 expression levels were significantly downregulated after 36 h of treatment with WT-E and its fractions, WT-H and WT-C (Figure 4A).

These results indicate that the induction of DNA double-strand breaks by WT extract triggers a coordinated suppressive response in the G1/S regulatory machinery, effectively stalling the cell cycle before the DNA synthesis phase.

### 2.4. WT Leaf Extract Inhibits the MAPK Pathway

**Figure 5:**
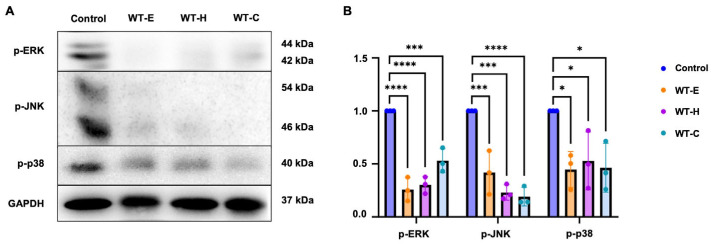
Suppression of the MAPK signaling pathway by WT leaf extract. (**A**) HT-29 cells were treated with respective IC_50_ value concentrations of WT extract and its fractions for 36 h. Protein levels of p-ERK, p-JNK and p-p38 levels were determined by Western blot analysis. (**B**) Densitometric quantification of band intensities using ImageJ software. GAPDH was used as the loading control. Data are expressed as the mean ± S.D. from three independent experiments. Statistical significance: * *p* < 0.05, *** *p* < 0.001 and **** *p* < 0.0001vs. control.

The mitogen-activated protein kinase signaling pathways play essential roles in regulating diverse cellular processes, including proliferation, differentiation, apoptosis, autophagy, and cell cycle arrest. To investigate whether the observed effects were mediated via these pathways, the phosphorylation levels of key MAPKs were evaluated by Western blotting (Figure 5). Treatment with WT-E and its solvent fractions, WT-H and WT-C markedly suppressed the expression levels of p-ERK1/2, p-p38 MAPK and p-JNK. These results suggest that WT extract inhibits the MAPK signaling pathway in HT-29 cells.

### 2.5. WT Leaf Extract Did Not Induce Apoptosis

**Figure 6:**
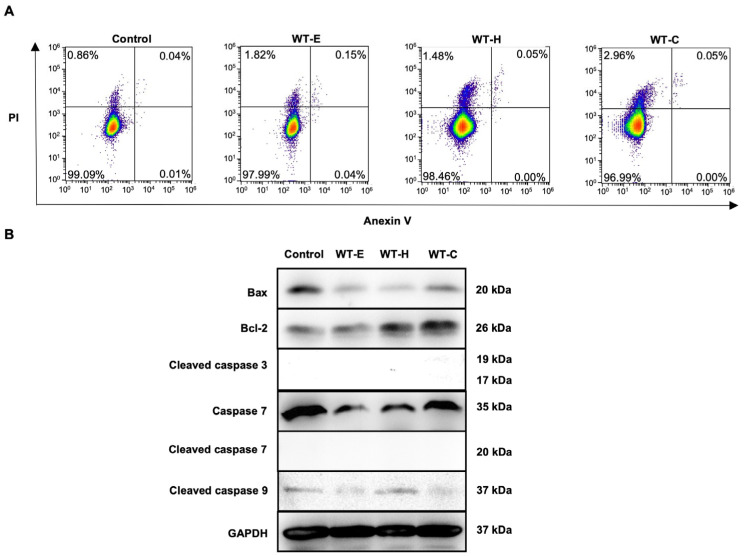
Assessment of apoptosis induction by WT leaf extracts in HT-29 cells. (**A**) Flow cytometric analysis of HT-29 cells following 36 h of treatment with WT-E and its solvent fractions at their respective IC_50_ concentrations. Cells were co-stained with Annexin V-FITC and propidium iodide (PI) to detect apoptotic and necrotic populations. (**B**) Western blot analysis of apoptosis-related proteins in HT-29 cells treated with WT-E and its fractions for 36 h. Representative blots show the expression levels of Bax and Bcl-2, and the proteolytic cleavage of caspase-3, -7, and -9. GAPDH was used as the loading control to ensure equal protein loading.

Apoptosis, a form of programmed cell death, maintains the homeostatic balance between cell proliferation and death. To further investigate the mechanisms by which WT leaf extract influences cancer cell growth, apoptosis-related assays were performed on HT-29 cells. Flow cytometric analysis revealed a significant increase specifically in PI-positive cells without a corresponding increase in Annexin V-positivity following treatment with WT leaf extracts. This lack of Annexin V/PI double-positivity suggests that the induced cell death is distinct from classical apoptosis (Figure 6A).

To definitively confirm whether the observed cell death was mediated by apoptosis, the expression of key pro-apoptotic markers was evaluated via Western blotting. The results demonstrated that neither the WT-E nor its solvent fractions induced the proteolytic cleavage of caspases-3, -7, or -9. Interestingly, treatment led to the downregulation of the pro-apoptotic protein Bax and a concomitant upregulation of the anti-apoptotic protein Bcl-2 (Figure 6B). This significant decrease in the Bax/Bcl-2 ratio further substantiates that the intrinsic mitochondrial apoptotic pathway remained inactive. Consequently, these findings provide robust evidence that WT treatment does not trigger classical apoptosis in HT-29 cells, indicating that the observed cytotoxicity is mediated by an alternative execution program, such as autophagic cell death.

### 2.6. WT Leaf Extract-Induced Autophagy in HT-29 Cells

**Figure 7:**
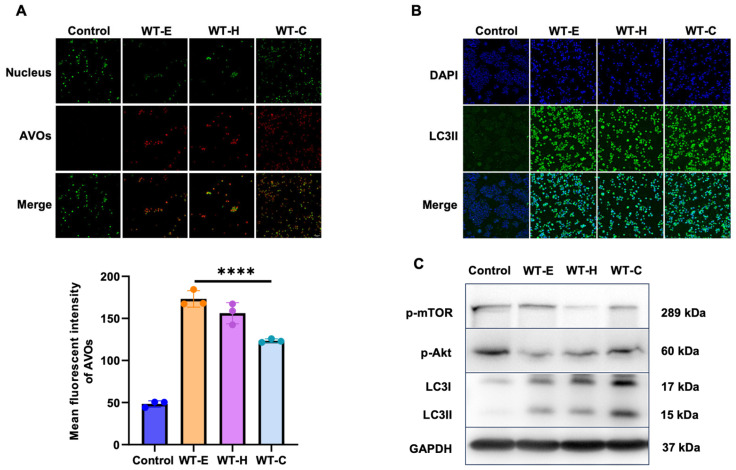
Induction of autophagy by WT leaf extracts in HT-29 cells. HT-29 cells were treated with WT-E and its solvent fractions at their respective IC_50_ concentrations for 36 h. (**A**) Detection of acidic vesicular organelles (AVOs) by acridine orange staining. Red fluorescence indicates AVOs, while green fluorescence represents the cytoplasm and nuclei (Scale bar = 100 μm). Red fluorescence intensities were quantified using ImageJ software. (**B**) Immunofluorescence analysis of LC3 expression. Cells were stained with anti-LC3 (FITC; green) and counterstained with DAPI (blue). Representative images were captured using confocal microscopy (Scale bar = 100 μm). (**C**) Western blot analysis of p-mTOR, p-Akt, and LC3-I/II protein levels. GAPDH was used as the loading control. Data represent the mean ± S.D. (*n* = 3). Statistical significance: **** *p* < 0.0001 vs. control.

Given that no classical apoptosis was detected, we investigated whether WT extract treatment induced autophagy in HT-29 cells. To evaluate autophagic activity, the formation of acidic vesicular organelles (AVOs) was examined using acridine orange staining. In this assay, the cytoplasm and nuclei emit bright green fluorescence, acidic compartments—such as lysosomes and autolysosomes—exhibit bright red fluorescence. The intensity of this red fluorescence correlates with the acidity and volume of AVOs, serving as a marker for autophagic progression. Treatment with WT leaf extract and its solvent fractions significantly promoted AVO formation at their respective IC_50_ concentrations (Figure 7A).

To determine whether the Akt/mTOR signaling pathway mediates this process, the phosphorylation levels of Akt and mTOR were evaluated by Western blotting. Following treatment with WT leaf extracts, a robust and sustained decrease in p-Akt and p-mTOR levels was observed (Figure 7C). These results suggest that WT leaf extracts inhibit HT-29 cell proliferation by suppressing Akt phosphorylation and mTOR kinase activity, thereby triggering autophagy. Furthermore, confocal immunofluorescence analysis revealed a significant increase in LC3-II-positive puncta (granules) in WT-treated cells compared to the untreated control (Figure 7B), further confirming the induction of autophagy.

### 2.7. Inhibition of Autophagy Suppressed WT Leaf Extract-Induced Cell Death in HT-29 Cells

**Figure 8:**
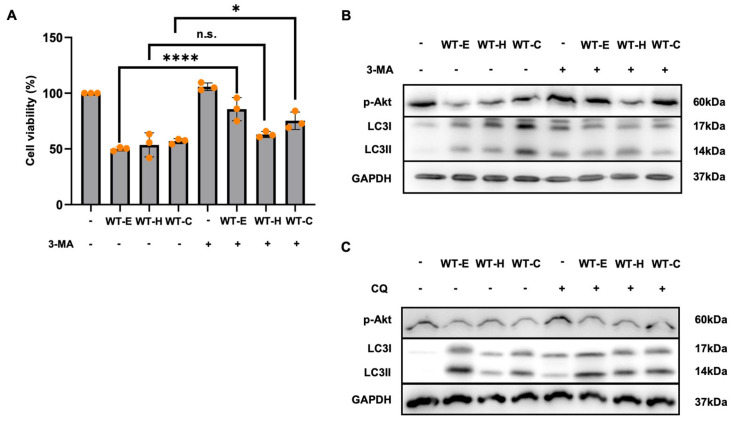
Inhibition of autophagy attenuates WT leaf extract-induced cell death in HT-29 cells. HT-29 cells were pretreated with or without 3-MA (100 μM) for 1 h, followed by treatment with WT-E, WT-H, or WT-C at their respective IC_50_ concentrations for 36 h. (**A**) Cell viability was measured using the CCK-8 assay. Data are expressed as the mean ± S.D. (*n* = 3). Statistical significance: * *p* < 0.05, **** *p* < 0.0001 vs. control; n.s., not significant. (**B**) Western blot analysis of phosphorylated Akt and LC3-I/II protein levels was performed. (**C**) HT-29 cells were treated with or without CQ (50 μM) for 3 h prior to cell collection. Band intensities were quantified using ImageJ software. GAPDH was used as the loading control.

To determine whether autophagy contributes to WT leaf extract-induced cell death, cell viability was assessed in the presence of 3-methyladenine (3-MA), a pharmacological inhibitor of early-stage autophagy. The anti-proliferative effects of WT-E and WT-C were significantly attenuated by 3-MA treatment (Figure 8A), suggesting that WT leaf extract-induced autophagy facilitates cell death rather than serving a cytoprotective role.

Consistent with these findings, Western blot analysis revealed that the elevated LC3-II levels induced by WT-C were markedly suppressed upon co-treatment with 3-MA, confirming the effective inhibition of autophagosome formation (Figure 8B). Furthermore, the downregulation of phosphorylated Akt by WT leaf extracts was partially restored in the presence of 3-MA, indicating a potential feedback loop between autophagic initiation and survival signaling.

To further validate autophagic flux, HT-29 cells were treated with chloroquine (CQ), a late-stage autophagy inhibitor that prevents lysosomal degradation. As shown in Figure 8C, co-treatment of WT-H and WT-C with CQ led to a further accumulation of LC3-II compared to treatment with the WT fractions alone. This result confirms that the WT fractions promote complete autophagic flux rather than impairing lysosomal function. Notably, p-Akt levels remained unchanged upon CQ addition, indicating that the suppression of the Akt signaling pathway occurs upstream of autophagosome–lysosome fusion. Collectively, these findings provide definitive evidence that WT extracts induce autophagy-mediated cell death in HT-29 cells by suppressing the Akt signaling axis.

### 2.8. WT Leaf Extract-Induced ROS Production

**Figure 9:**
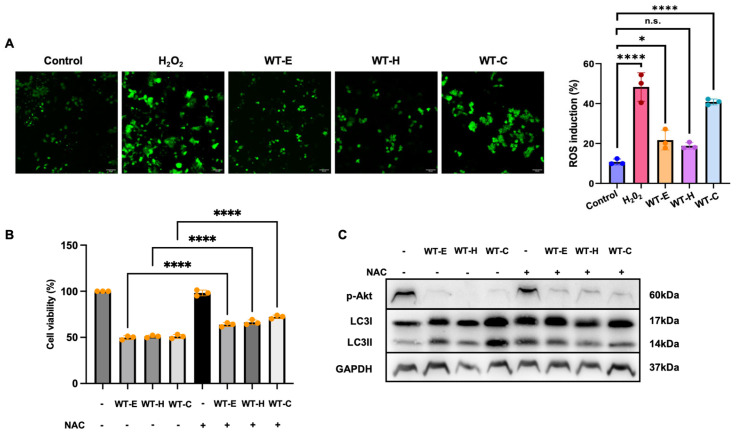
Role of intracellular ROS generation in WT extract-induced autophagic cell death in HT-29 cells. HT-29 cells were treated with WT-E, WT-H, and WT-C at their respective IC_50_ concentrations for 36 h. (**A**) Representative fluorescence microscopy images followed by staining with DCFH-DA (left). Quantitative analysis of relative fluorescence intensity to evaluate ROS production (right). H_2_O_2_ (10 μM) was employed as a positive control. (**B**,**C**) Rescue experiments using ROS scavenger *N*-accetylcystein (NAC). HT-29 cells were pre-treated with or without NAC (10 mM) for 3 h, followed by adding WT-E, WT-H, or WT-C at their respective IC_50_ concentrations for 36 h. (**B**) Cell viability was measured using the CCK-8 assay. (**C**) Western blot analysis of phosphorylated Akt (p-Akt) and LC3-I/II protein levels. GAPDH was used as the loading control. Data are expressed as the mean ± S.D. (*n* = 3). Statistical significance: * *p* < 0.05 and **** *p* < 0.0001 vs. control; n.s., not significant.

Reactive oxygen species (ROS) play a crucial role in signal transduction pathways that regulate cell growth, differentiation, and cellular redox homeostasis [14]. To evaluate ROS generation, HT-29 cells were loaded with the cell-permeable probe DCFH-DA following a 36 h treatment with WT leaf extracts. As shown in Figure 9A, treatment with WT leaf extract—particularly the chloroform fraction (WT-C)—significantly induced ROS production compared to the untreated control. These results suggest that the induction of oxidative stress may be a key mechanism underlying the anti-proliferative effects of the WT extract. To further investigate the causal relationship between ROS generation and WT-induced cell death, rescue experiments were performed using the antioxidant *N*-acetylcysteine (NAC). As illustrated in Figure 9B, pretreatment with NAC significantly restored cell viability, effectively attenuating the cytotoxic effects of WT extracts. Furthermore, Western blot analysis revealed that NAC treatment successfully reversed the inhibition of p-Akt and suppressed the conversion of LC3-I to LC3-II induced by the extracts (Figure 9C). Notably, the most pronounced recovery was observed in the WT-C-treated group. These results indicate that the induction of oxidative stress is a critical upstream mechanism underlying the Akt/mTOR-mediated autophagic cell death triggered by WT extracts.

### 2.9. GC-MS Analysis of WT-H and WT-C Fractions

**Figure 10:**
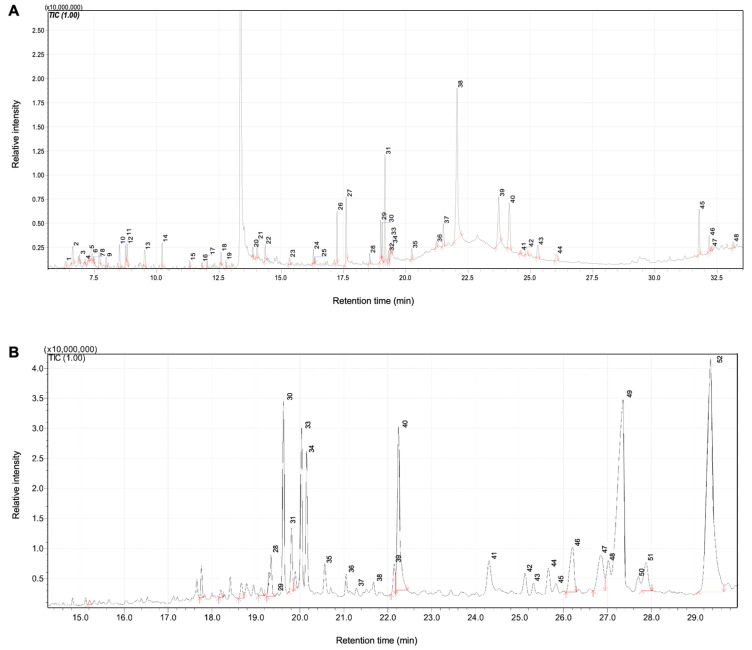
GC chromatograms of WT-H (**A**) and WT-C (**B**).

**Figure 11:**
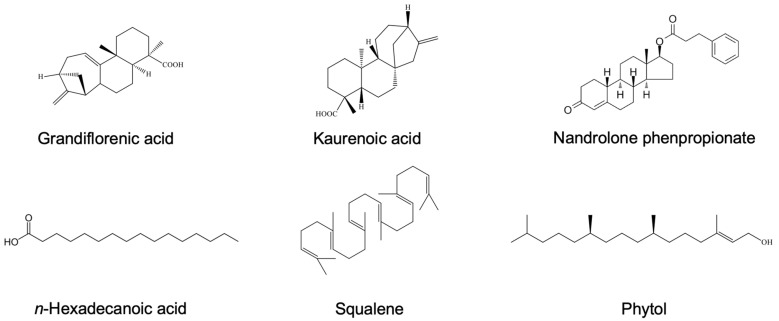
Chemical structures of the main compounds in WT-H.

GC-MS analysis of the WT-H and WT-C fractions revealed a diverse profile of chemical constituents (Figure 10). The numbers marked on the chromatogram peaks correspond to the specific bioactive compounds identified via GC-MS. A complete list of these numbered constituents, along with their retention times and peak areas, is provided in Tables S1 and S2. Detailed examination of the chromatograms and mass-to-charge (*m*/*z*) ratios confirmed the presence of various lipophilic metabolites in WT-H, primarily consisting of diterpenes and fatty acid esters. As illustrated in Figure 11, the key compounds identified in WT-H included: grandiflorenic acid (retention time [RT]: 22.06 min, relative intensity: 25.71%), kaurenoic acid (RT: 23.73 min, 7.78%), phytol (RT: 19.16 min, 7.34%), and nandrolone phenpropionate (RT: 24.15 min, 6.8%). Other notable constituents included n-hexadecanoic acid (RT: 17.60 min, 4.95%), squalene (RT: 31.77 min, 4.35%), hexadecanoic acid methyl ester (RT: 17.24 min, 3.4%), and 9,12-octadecadienoic acid (Z,Z)-methyl ester (RT: 18.99 min, 2.51%).

**Figure 12:**
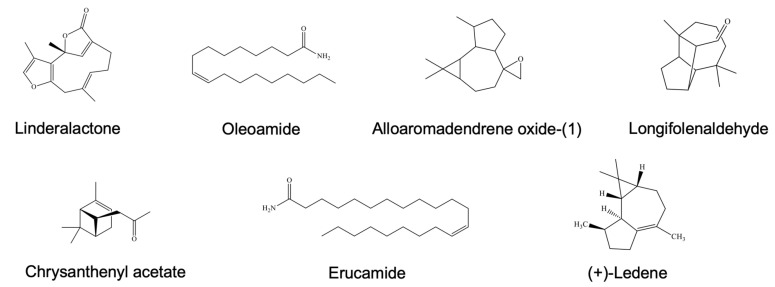
Chemical structures of the main compounds in WT-C.

Similarly, the WT-C fraction was characterized by several major components, as shown in Figure 12. These included linderalactone (RT: 29.34 min, 25.37%), ledene (RT: 27.35 min, 20.11%), oleamide (RT: 22.24 min, 8.11%), alloaromadendrene oxide-(1) (RT: 26.2 min, 5.68%), chrysanthenyl acetate (RT: 26.84 min, 3.6%), longifolenaldehyde (RT: 19.34 min, 3.42%), and erucamide (RT: 24.30 min, 2.72%).

## 3. Discussion

Colorectal cancer remains one of the most prevalent malignancies globally. The inherent toxicity and adverse effects of conventional treatments significantly impact both the therapeutic efficacy and the prognosis of patient populations. Consequently, identifying safe and efficient bioactive compounds from traditional medicinal plants represents a strategic approach for developing novel anticancer therapies.

While the crude ethanol extract (WT-E) exhibited a relatively low selectivity index (S. I. < 2), further solvent partitioning significantly enhanced the safety profile of the resulting bioactive fractions. Specifically, the *n*-hexane (WT-H) and chloroform (WT-C) fractions demonstrated superior selectivity when tested against OUMS-36 normal fibroblast cells, yielding S. I. values of 2.09 and 7.08, respectively. This marked improvement suggests that fractional purification effectively concentrates anti-cancer metabolites while concurrently reducing non-specific cytotoxicity. These findings indicate that the refined WT-H and WT-C fractions possess favorable safety margins, supporting their potential for further therapeutic development. Further validation using normal colonic epithelial cells, such as NCM460, would be beneficial to evaluate their tissue-specific selectivity more precisely in future studies.

We demonstrated that *Wedelia trilobata* (WT) extract inhibits cell proliferation by inducing G1/S phase arrest and suppressing the migratory activity of CRC cells. Furthermore, our findings suggest that the anticancer effects of the WT extract are mediated through the modulation of the MAPK/ERK and Akt/mTOR signaling pathways. Notably, WT treatment induced significant DNA damage, as evidenced by elevated levels of the DNA damage marker γ-H2AX.

It is well-established that many anticancer agents induce metabolic stress in cancer cells, resulting in excessive generation of reactive oxygen species (ROS). The subsequent oxidative stress leads to the accumulation of misfolded proteins, which in turn triggers cellular stress responses such as autophagy or apoptosis [15]. Differentiating between Type I programmed cell death (apoptosis) and Type II (autophagy) is essential for elucidating the pharmacological mechanisms of natural extracts. While apoptosis is conventionally defined by caspase cascade activation and cellular fragmentation, autophagy-mediated cell death is characterized by the accumulation of double-membrane autophagosomes and the lipidation of LC3-I to LC3-II. The molecular crosstalk between these two pathways remains a complex area of oncology; they do not function in isolation but rather undergo a sophisticated interplay, often acting in a coordinated or mutually inhibitory manner depending on the level of cellular stress [16]. Consistent with this framework, our findings suggest that the anticancer effects of WT extract extend beyond traditional apoptosis. This is evidenced by the observation that apoptotic rates did not correlate with the overall decrease in cell survival. Classically, commitment to apoptosis is governed by the intricate balance between pro-apoptotic (Bax) and anti-apoptotic (Bcl-2) members of the Bcl-2 family. An increase in the Bax/Bcl-2 ratio typically leads to mitochondrial outer membrane permeabilization and subsequent caspase activation [17]. However, our results revealed a paradoxical downregulation of Bax together with upregulation of Bcl-2 following WT treatment. This atypical shift in the Bax/Bcl-2 ratio suggests a robust, albeit transient, compensatory survival response by HT-29 cells, potentially representing an attempt to counteract the severe oxidative stress and Akt inhibition triggered by the extract. In many cancer models, the upregulation of Bcl-2 serves as a cytoprotective shield against mitochondrial apoptosis; nevertheless, our data indicate that this defensive mechanism was insufficient to avert cellular demise. Instead, the persistent “oxidative burst” and the sustained collapse of the Akt/mTOR survival axis effectively circumvented the apoptotic machinery, redirecting cellular fate toward an irreversible autophagic execution program. While morphological features such as nuclear condensation were observed, the lack of caspase-3, -7, and -9 cleavage further reinforces that WT-induced cytotoxicity in HT-29 cells is a caspase-independent process, distinguishing it from classical Type I programmed cell death.

The induction of autophagy is fundamentally characterized by the formation of acidic vesicular organelles (AVOs). While AVOs can initially serve a cytoprotective role by maintaining pH homeostasis and providing catabolites for cellular recovery, their excessive accumulation often transitions into a pro-death signal, leading to non-apoptotic programmed cell death [18]. In the present study, acridine orange (AO) staining confirmed a significant increase in AVO formation in HT-29 cells treated with all three WT extract fractions. This was further validated by Western blot analysis, which demonstrated a marked increase in LC3-II expression alongside a concomitant decrease in the phosphorylation of Akt and mTOR.

The use of 3-MA, a class III PI3K inhibitor, was pivotal in elucidating the functional role of autophagy in our model. The significant rescue of cell viability upon 3-MA treatment confirms that WT-induced autophagy does not function as a typical cytoprotective stress response—often observed during nutrient starvation or moderate oxidative stress—but rather acts as a terminal execution mechanism (autophagy-mediated cell death). This transition is specifically driven by the potent suppression of the Akt/mTOR axis, which redirects the autophagic machinery from a survival effort toward a pro-death pathway in HT-29 cells. To determine whether the elevated LC3-II levels resulted from the induction of autophagy or the inhibition of autophagosome clearance, we monitored the autophagic flux using chloroquine (CQ), a lysosomotropic agent that blocks the fusion of autophagosomes with lysosomes [19]. Our results demonstrated that co-treatment with WT fractions and CQ led to a further accumulation of LC3-II compared to treatment with the fractions alone. This synergistic effect definitively indicates that WT extracts do not impair lysosomal function but instead significantly accelerate the rate of autophagic sequestration and turnover in HT-29 cells. Notably, the observation that p-Akt levels remained suppressed even in the presence of CQ further confirms that the inhibition of Akt/mTOR survival axis is an upstream regulatory event, occurring independently of the downstream lysosomal degradation stage. Collectively, these findings reinforce the conclusion that *W. trilobata* leaf extract triggers a complete and functional autophagic program that ultimately culminates in cell death.

Beyond autophagy, the MAPK signaling pathway plays a central role in colorectal cancer progression by regulating differentiation, motility, and survival [20]. The JNK and p38 MAPK pathways are primarily involved in cellular stress responses and apoptosis, while the ERK signaling pathway predominantly regulates cell proliferation and growth [21]. The PI3K/Akt/mTOR pathway also regulates cell proliferation, growth, cell cycle progression, angiogenesis, and motility [22]. Some components identified in the WT hexane and chloroform fractions by GC-MS in our study have been reported previously to possess significant anticancer activities via autophagy and/or apoptosis. For instance, linderalactone has been shown to induce cell cycle arrest and apoptosis via the PI3K/Akt [23] and the JAK/STAT pathways [24]. Similarly, oleamide has been reported to trigger both apoptosis and autophagy within 48 h in other cancer models [25,26]. Chrysanthenyl acetate, a main component in *Tanacetum sinaicum* leaf, has been reported to induce autophagy, inhibit cell migration, and cause DNA damage in the HeLa and MCF7 cancer cell lines [27]. Furthermore, the diterpenes kaurenoic acid and grandiflorenic acid, which are hallmark compounds of WT leaf extract, are known to stimulate oxidative stress via the induction of ROS generation, leading to G1 arrest and dual-pathway cell death (apoptosis and autophagy) [12,28]. A critical feature of these diterpenes is their ability to induce oxidative stress, which acts as a common upstream trigger for both cell death pathways. *n*-Hexadecanoic acid was reported as an effective agent against colorectal cancer cells in vitro by inducing ROS and G0/G1 cell cycle arrest [29]. On the other hand, a distinctive finding in our study on HT-29 cells is that the WT leaf extract primarily drove a pro-autophagic response. HT-29 cells treated with WT extract did not show the typical hallmarks of apoptosis, although some components are known to induce apoptosis in other cancer cells. This suggests that the specific concentrations or the unique synergistic environment of these compounds within our WT extract selectively favor autophagic cell death over apoptotic pathways in the colorectal cancer context.

The collective generation of ROS by WT metabolites likely reaches a specific threshold that dictates the mode of programmed cell death. Under normal physiological conditions, basal ROS levels maintain homeostasis, while moderate oxidative stress typically triggers cytoprotective autophagy as a survival mechanism via AMPK activation or direct oxidation of Atg4 [30,31]. However, our findings demonstrate that WT extract subjects HT-29 cells to excessive oxidative stress. Upon reaching this critical threshold of ROS accumulation, cellular signaling appears to transition from a cytoprotective response to programmed cell death. While extreme ROS levels often trigger mitochondrial-mediated apoptosis in various cancer models, the current study reveals a distinct mechanism in HT-29 cells. This potent oxidative burst effectively circumvents the classical apoptotic pathway, instead driving the cells toward an irreversible autophagic execution program [32]. The pivotal role of ROS in this process was further confirmed by our rescue experiments; scavenging ROS with NAC not only restored cell viability but also reversed the inhibition of p-Akt and the subsequent induction of autophagy. This confirms a hierarchical signaling axis where ROS serves as the primary initiator that suppresses the Akt/mTOR signaling pathway to execute autophagic death. In the specific genetic context of HT-29 cells, the persistent oxidative stress leads to the sustained inhibition of the Akt/mTOR survival axis. Consequently, the cell is ‘locked’ into a terminal autophagic state, directly contributing to cell death rather than survival [33,34].

The predominance of autophagy-mediated cell death over apoptosis observed in our study provides novel insight into the pharmacological potential of WT leaf extract. While previous research demonstrated that WT methanol extract induces apoptosis in human leukemia cells through BCR/ABL and Akt suppression [13], our findings reveal a distinct downstream response in HT-29 colorectal cancer cells. This discrepancy likely arises from the divergent genetic landscapes of these cancer models. Whereas certain leukemia cells are often primed for apoptosis, the *p53*-mutant status of HT-29 cells is frequently associated with an ‘apoptotic block,’ which may favor transition toward terminal autophagy under conditions of severe oxidative stress. For example, oridonin, a natural diterpenoid, has been shown to induce autophagy-mediated death in HT-29 cells by suppressing the Akt/mTOR pathway, a mechanism that aligns with our observations [35]. Similarly, research involving piperlongumine [36] suggests that when the *p53*-dependent apoptotic machinery is impaired, the accumulation of ROS serves as an alternative stimulus for initiation of pro-death autophagy. Our results reinforce this model, suggesting that the WT extract exploits this specific vulnerability. This *p53*-independent mechanism suggests that WT-derived constituents may be particularly effective against apoptosis-resistant tumors that have lost *p53* function. Although further investigation using *p53*-wild-type models, such as HCT116, is required to determine the full spectrum of its generalizability, the current findings establish a robust proof-of-concept for using *W. trilobata* to target recalcitrant CRC through non-canonical death programs. While further comparative studies using *p53* wild-type models would help delineate the exact regulatory role of *p53*, the current data demonstrate that WT extract can effectively bypass apoptotic resistance in *p53*-deficient colorectal cancer cells via the ROS-Akt-autophagy axis.

A key finding of this study is the comparable anticancer potency of the WT-H and WT-C fractions despite their differing chemical compositions. While previous literature has largely attributed the bioactivity of *W. trilobata* to grandiflorenic acid [12], our data demonstrate that the WT-C fraction remains highly effective in inducing autophagy-mediated death in HT-29 cells even in the absence of this specific compound. This suggests that the observed anti-tumor activity arises either from synergistic effects or from the presence of alternative potent metabolites, such as linderalactone, in the chloroform fraction. Furthermore, the ability of both fractions to target the same Akt/mTOR signaling axis—regardless of their primary constituents—suggests that different classes of terpenoids in WT leaves may converge on the same molecular targets. This ‘multi-constituent, multi-target’ characteristic is a hallmark of botanical therapeutics and underscores the importance of evaluating bioactive fractions rather than focusing solely on isolated compounds. Such a holistic mechanism may enhance therapeutic efficacy by simultaneously modulating multiple nodes within a survival pathway, potentially reducing the likelihood of cellular resistance. While the present study establishes the pharmacological potential of these phytocomplexes, future research involving the isolation and parallel testing of pure grandiflorenic acid and linderalactone will further delineate their individual versus collective contributions.

In the present study, involvement of autophagy in WT-induced cell death was supported using 3-MA, a widely recognized pharmacological inhibitor of the class III PI3K/Vps34 complex. We observed that 3-MA partially rescued the viability of HT-29 cells, suggesting that autophagy plays a pro-death role in this context. However, we acknowledge the inherent limitations of 3-MA, such as potential off-target effects on other PI3K-related signaling pathways. To address this, we employed a multi-parametric approach－incorporating ROS-scavenging assays (NAC) and autophagic flux monitoring (chloroquine)－to provide robust evidence for the ROS-Akt-Autophagy axis. While these in vitro findings delineate a clear molecular hierarchy, we recognize that further genetic interventions, such as the silencing of *ATG5* or *BECN1* genes, would be valuable to further refine the molecular specificity of this cell death program. Furthermore, subsequent in vivo investigations using HT-29 xenograft models are warranted to validate the systemic efficacy and safety of WT extracts. Such studies will be instrumental in determining how the ROS-driven autophagic signaling observed here translates into tumor growth inhibition within a complex physiological environment. This study thus provides the essential mechanistic foundation required for the future development of *W. trilobata*-derived metabolites as potential therapeutic agents for colorectal cancer.

In conclusion, this study demonstrates that the *Wedelia trilobata* (WT) ethanol extract exerts potent anti-tumor activity by exploiting signaling vulnerabilities of HT-29 colorectal cancer cells. Our findings establish that the extract induces intracellular ROS generation beyond a critical threshold, effectively suppressing the Akt/mTOR survival axis. This oxidative stress-driven signaling shift redirects the autophagic machinery from a cytoprotective role toward a terminal pro-death execution program. Notably, in this colorectal cancer model, the transition to autophagic cell death occurs independently of classical apoptosis. These results position *Wedelia trilobata* (L.) Hitchc. as a promising therapeutic candidate, offering a selective strategy to eliminate recalcitrant colorectal cancer cells by disrupting the delicate homeostasis between oxidative stress and survival signaling.

## 4. Materials and Methods

### 4.1. Cell Line and Cell Culture

All cell lines were purchased from the JCRB Cell Bank (National Institutes of Biomedical Innovation, Health and Nutrition, Osaka, Japan). The human cancer cell lines used in this study included colorectal adenocarcinoma HT-29 (No. JCRB1383), cervical carcinoma HeLa (No. JCRB9004), hepatocellular carcinoma HepG2 (No. JCRB1592) and Huh7 (No. JCRB0403), and lung carcinoma A549 (No. JCRB0076). Human embryonic kidney 293 cells (HEK293) (No. JCRB9068) and human normal embryo fibroblast cells (OUMS-36) (No. JCRB1006.0) were employed as normal controls. The cells were maintained according to the manufacturer’s instructions in either Eagle’s Minimum Essential Medium (EMEM; Nacalai Tesque, Kyoto, Japan) or Dulbecco’s Modified Eagle Medium (DMEM; Fujifilm Wako Pure Chemical, Osaka, Japan). Both media were supplemented with 10% fetal bovine serum and 1% penicillin–streptomycin. Cultures were incubated at 37 °C in a humidified atmosphere containing 5% CO_2_. The following reagents were used in this study: *N*-acetylcysteine, chloroquine diphosphate salt and 3-methyladenine were purchased from Sigma-Aldrich (St. Louis, MO, USA).

### 4.2. Preparation of Wedelia trilobata (L.) Extract and Its Solvent Fractions

The leaves of *Wedelia trilobata* (L.) Hitchc. were collected from Can Tho, Vietnam. The plant species was identified and authenticated by experts at the Vietnam Academy of Agriculture Sciences, Plant Resources Center. A voucher specimen (VNM00122021CTU) was deposited at the department’s herbarium for future reference.

The crude ethanol extract of *W. trilobata* (WT-E) was prepared and provided by the Faculty of Chemistry, College of Natural and Sciences, Can Tho University. Briefly, the dried leaf powder was extracted with ethanol, and the solvent was subsequently removed using a rotary evaporator under reduced pressure to yield 25 g of concentrated extract. The WT-E was stored at −20 °C until further use for solvent fractionation and biological assays. For solvent partitioning, 20 g of WT-E was sequentially fractionated into *n*-hexane (WT-H), chloroform (WT-C), ethyl acetate (WT-EA), and water (WT-W) fractions. Each fraction was concentrated using a rotary evaporator (Advantec, Tokyo, Japan) at 45 °C, followed by freeze-drying. The resulting fractions were stored at 4 °C until further analysis.

### 4.3. Cell Viability and Colony Formation Tests

Stock solutions of WT-E and its solvent fractions were prepared at 4 mg/mL in 5% (*v*/*v*) dimethyl sulfoxide (DMSO; FUJIFILM-Wako, Osaka, Japan) and subsequently diluted to target concentrations using DMEM.

Cell viability was evaluated via the CCK-8 assay. HT-29 cells (5 × 10^4^ cells/well) and HEK293 cells (10^5^ cells/well) were seeded in 96-well plates and allowed to adhere overnight. The cells were then treated with serial two-fold dilutions of the WT samples for 48 h, while ensuring that the final DMSO concentration remained at 0.5% (*v*/*v*). Afterward, the medium was replaced with CCK-8 reagent-containing medium (Dojindo Laboratories, Kumamoto, Japan) and incubated for 3 h. The absorbance at 450 nm was recorded using an SH-1200 microplate reader (Corona Electric, Hitachinaka, Japan). IC_50_ values, defined as the concentrations required to achieve 50% growth inhibition, were calculated from the dose–response curves. The selectivity index (S. I.) was determined as the ratio of the IC_50_ for normal cells to that for HT-29 cells.

For the clonogenic potential assessment, HT-29 cells (100 cells/well) were seeded in 6-well plates and maintained at 37 °C with 5% CO_2_. Following initial attachment, the cells were exposed to DMEM containing WT-E or its fractions at their respective IC_50_ concentrations for 48 h. Subsequently, the treatment medium was replaced with fresh DMEM, which was replenished every 2–3 days. On day 10, the resulting colonies were fixed with 4% paraformaldehyde at 4 °C for 15 min and visualized using 0.5% (*w*/*w*) crystal violet staining. Cell clusters comprising at least 50 individual cells were identified as colonies and manually counted under an Olympus stereomicroscope (Tokyo, Japan).

### 4.4. Cell Migration Test

The wound healing assay was employed to evaluate the effects of WT samples on cell migration. HT-29 cells (3 × 10^5^ cells/well) were seeded in 24-well plates. After approximately 3 days, once the cells reached confluence, the medium was replaced with fresh medium containing mitomycin C (10 µg/mL) for 2 h to inhibit cell proliferation, ensuring that wound closure resulted solely from cell migration. A linear scratch was then created across the center of each well using a sterile 200 µL pipette tip. The wells were washed three times with PBS to remove detached cells and debris. Subsequently, DMEM containing WT-E or its solvent fractions at their respective IC_50_ concentrations was added to each well. Images of the scratch area were captured at 0 and 24 h post-scratching. The width of the scratch was quantified using image analysis by ImageJ software (version 1.54j; National Institutes of Health, Bethesda, MD, USA), and the migration distances were calculated accordingly.

### 4.5. Quantitative RT-PCR (RT-qPCR)

HT-29 cells were treated with WT-E or its solvent fractions prior to total RNA extraction using the miRNeasy Kit (Qiagen, Hilden, Germany). The concentration and quality of isolated RNA were evaluated using a NanoDrop spectrophotometer (Thermo Fisher Scientific, Wilmington, DE, USA). Subsequently, 1 µg of total RNA was utilized for cDNA synthesis through reverse transcription, employing the Transcriptor Universal cDNA Master Kit (Sigma-Aldrich, USA) according to the manufacturer’s instructions. qPCR was performed using the FastStart SYBR Green Master Kit (Sigma-Aldrich, USA) with 50 ng of cDNA as template and gene-specific primers (Thermo Fisher Scientific, USA). The primer sequences were as follows: *PCNA* (proliferating cell nuclear antigen), forward 5′-TTTGGTGCAGCTCACCCTG-3′ and reverse 5′-CGCGTTATCTTCGGCCCTTA-3′; and *GAPDH* (glyceraldehyde 3-phosphate dehydrogenase), forward 5′-CCTCAAGATCATCAGCAATGCC-3′ and reverse 5′-ACAGTCTTCTGGGTGGCAGT-3′. Amplification was performed using a LightCycler 96 System (Roche Diagnostics, Mannheim, Germany). Relative gene expression levels were calculated using the 2^−ΔΔCt^ method, with *GAPDH* as the internal reference control.

### 4.6. Western Blotting

HT-29 cells were seeded in 6-well plates and exposed to various concentrations of WT-E or its bioactive fractions for a duration of 36 h. Total cellular protein was isolated using RIPA lysis buffer (FUJIFILM Wako, Japan) containing a protease and phosphatase inhibitor cocktail. Protein quantification was performed using the Pierce™ BCA protein assay kit (Thermo Fisher Scientific, USA). For protein separation, equal amounts of lysates were loaded onto 15% SDS-PAGE gels and subsequently transferred onto polyvinylidene fluoride (PVDF) membranes. The membranes were blocked for 1 h with 5% skim milk (FUJIFILM Wako) and then incubated overnight at 4 °C with specific primary antibodies, including: mouse anti-γH2AX (phospho-Ser139; BioLegend, San Diego, CA, USA), rabbit anti-cyclin E (Proteintech, Rosemont, IL, USA), mouse anti-CDK2, Bcl-2 (Santa Cruz Biotechnology, Dallas, TX, USA), and rabbit antibodies LC3A/B, p-JNK, p-p38, p-ERK1/2, p-Akt, p-mTOR, cleaved caspase-3, cleaved caspase-7, cleaved caspase-9, Bax and GAPDH (Cell Signaling Technology, Danvers, MA, USA). All antibodies were prepared at dilutions recommended by the manufacturers. Following three rinses in Tris-buffered saline with 0.5% Tween 20 (TBS-T), the membranes were treated with suitable HRP-conjugated secondary antibodies (Cell Signaling Technology) in TBS-T containing 5% bovine serum albumin (BSA fraction V; Roche, Mannheim, Germany) for 1 h at room temperature. Protein signals were detected using ECL Select Western Blotting Detection Reagent (GE Healthcare, Pittsburgh, PA, USA) and captured via an AE-9300H Ez-Capture MG imaging system (ATTO Corp., Tokyo, Japan). The intensity of the protein bands was analyzed using ImageJ software (NIH, USA), with GAPDH serving as the internal loading control for normalization.

### 4.7. 7-AAD Staining

HT-29 cells were cultured on 4-well chamber slides (Nunc™ Lab-Tek^®^; Thermo Fisher Scientific, USA). Following treatment, the cells were washed twice with PBS and stained with 7-aminoactinomycin D (7-AAD; Molecular Probes, Eugene, OR, USA) for 30 min in the dark at room temperature. The samples were then mounted using Vectashield mounting medium (Vector Laboratories, Japan) and examined under a fluorescence microscope (FV10i; Olympus, Japan). The fluorescence intensity was quantified using ImageJ software (NIH, USA).

### 4.8. Intracellular ROS Levels Measurement

Intracellular reactive oxygen species (ROS) levels were determined using a photo-oxidation-resistant DCFH-DA probe (ROS Assay Kit; Dojindo, Kumamoto, Japan). HT-29 cells were treated with WT-E and its solvent fractions for 36 h. As a positive control, cells were incubated with 10 μM hydrogen peroxide (H_2_O_2_) for 30 min at 37 °C prior to harvesting. Following treatment, the cells were washed with phosphate-buffered saline (PBS) and incubated with 10 μM DCFH-DA at 37 °C for 30 min in the dark. After incubation, the cells were washed again with PBS to remove the extracellular probe. ROS-positive cells, characterized by green fluorescence, were visualized using an FV10i fluorescence microscope (Olympus, Tokyo, Japan). The mean fluorescence intensity was quantified using ImageJ software (NIH, USA). To evaluate the functional role of ROS, HT-29 cells were pre-treated with pharmacological inhibitors before extract exposure. For ROS scavenging, cells were incubated with 10 mM *N*-acetylcysteine (NAC) for 3 h prior to treatment with WT extracts.

### 4.9. Autophagic Flux

To monitor autophagic flux, cells were co-treated with 50 μM chloroquine diphosphate salt (CQ) during the 3 h incubation period. Following treatment, cell viability was assessed via CCK8 assay and protein expression was analyzed by Western blotting as described in Section 4.6.

### 4.10. AVO Staining

To visualize the formation of acidic vesicular organelles (AVOs), a hallmark of autophagy, HT-29 cells were stained with acridine orange (AO; Sigma-Aldrich, MO, USA). Briefly, after 36 h of incubation with the WT samples, the cells were subjected to AO staining (5 μM) to detect acidic compartments. The development of AVOs was observed using an FV10i fluorescence microscope (Olympus, Tokyo, Japan). Red fluorescence intensity—indicating the accumulation of AO in acidic vesicles—was captured and subsequently quantified using ImageJ software.

### 4.11. Immunocytochemistry

Immunofluorescence analysis was conducted by seeding HT-29 cells into eight-well chamber slides (Nunc™ Lab-Tek^®^, Thermo Fisher Scientific). Post-treatment with WT extract and fractions, a 4% paraformaldehyde phosphate-buffered solution (FUJIFILM-Wako, Japan) was applied for cell fixation. The localization of LC3A/B (LC3-I/II) was detected using a rabbit primary antibody (1:500) and an Alexa Fluor™ 594-labeled anti-rabbit IgG (1:800; Molecular Probes). For nuclear visualization, cells were stained with DAPI (Molecular Probes) for a duration of 30 min. After mounting with Vectashield (Vector Laboratories, Tokyo, Japan), samples were examined under an Olympus FV10i fluorescence microscope. ImageJ (NIH, USA) was utilized for the subsequent analysis of fluorescence intensity.

### 4.12. Flow Cytometry

To measure WT extract-induced apoptosis, an annexin V-FITC/PI Apoptosis detection kit (#640,914, Biolegend, San Diego, CA, USA) was used to quantify apoptotic cell death following the manufacturer’s instructions. HT-29 cells (both at 2 × 10^5^ cells/well) were seeded in 6-well plates overnight and treated with IC_50_ concentrations of WT-E, WT-H and WT-EA for 36 h before collection. Following treatment, HT-29 cells were detached with trypsin and rinsed once in Staining Buffer (Biolegend, USA). The cell pellets were then resuspended in the kit’s Binding Buffer and subjected to dual staining with Annexin V and propidium iodide solution for 15 min at 25 °C. Fluorescent signals were captured via an RF-500 flow cytometer (Sysmex, Hyogo, Japan). For the final analysis of cell death distribution, FCSalyzer 0.9.22 software was utilized to process the raw flow cytometry data.

### 4.13. GC-MS Analysis

The chemical compositions of the *n*-hexane and chloroform fractions of WT-E were analyzed using gas chromatography–mass spectrometry (GC-MS; GCMS-QP2020 NX, Shimadzu Corp., Kyoto, Japan). Gas chromatographic separation was performed using an Rtx-5MS capillary column (30 m × 0.25 mm i.d., 0.25 µm film thickness; Restek, Bellefonte, PA, USA). High-purity helium (≥99.999%) served as the carrier gas, maintained at a steady flow rate of 1.0 mL/min. The thermal gradient for the oven was programmed as follows: starting at an initial temperature of 60 °C (0 min hold), followed by a linear increase of 10 °C/min until reaching a final temperature of 250 °C, which was then maintained for an additional 10 min and finally increased at 15 °C/min to 300 °C (held for 3 min). The MS was operated in electron ionization (EI) mode with a full-scan range of *m*/*z* 50–500. Other parameters included splitless injection at 250 °C and an interface temperature of 250 °C. The chemical constituents were identified by comparing their mass spectra with those in the National Institute of Standards and Technology (NIST-14, Gaithersburg, MD, USA) and Wiley (Wiley Science Solutions, Hoboken, NJ, USA) spectral databases.

### 4.14. Statistical Analysis

Experimental data are presented as the mean ± standard deviation (SD). All statistical evaluations were conducted using GraphPad Prism 9.0 software (GraphPad Software, Inc., San Diego, CA, USA). Significant differences between groups were determined via one-way analysis of variance (ANOVA), followed by Dunnett’s post hoc test. Statistical significance was defined at *p*-values of <0.05, <0.01, <0.001, and <0.0001.

## Data Availability

All data generated or analyzed during this study are included in this published article and its Supplementary Materials. Raw datasets supporting the reported results are available from the corresponding author upon reasonable request.

## Supplementary Materials

The following supporting information can be downloaded at: https://www.mdpi.com/article/10.3390/ijms27104636/s1.

## Abbreviations

The following abbreviations are used in this manuscript:

Akt	Protein kinase B
CQ	Chloroquine
ERK	Extracellular signal-regulated kinase
G1 phase	The first phase of the cell cycle
JNK	c-Jun N-terminal kinases
mTOR	Mechanistic target of rapamycin
NAC	N-acetylcysteine
p-	phosphorylated
p38	p38 mitogen-activated protein kinase
p53	Tumor suppressor protein
S phase	The phase of DNA synthesis in the cell cycle
