# Supplementary material for: Wedelia trilobata (L.) Leaf Extract Induces Autophagy-Mediated Cell Death in HT-29 Colorectal Cancer Cells via Suppression of the Akt/mTOR Signaling Axis
Source: Int J Mol Sci. 2026 May 21;27(10):4636. doi: 10.3390/ijms27104636 (PMC13207333; doi:10.3390/ijms27104636)
Supplement: Supplementary file 1 [file ijms-27-04636-s001.zip › ijms-4282937-supplementary.pdf]

Supplementary Materials

Table S1. GC-MS analysis of *Wedelia triolobata* (L.) hexane fraction

Peak number	Retention time, min	Area, %	Compound names
1	6.343	0.69	*4,5-Diethyloctane*
2	6.635	1.9	5,6-Dimethyldecane
3	6.906	0.35	5,6-Dimethyldecane
4	7.118	0.31	2,4-dimethylundecane
5	7.263	0.25	3-ethyl-5-methyl-1-propylcyclohexane
6	7.409	0.57	1,1,3-Trimethylcyclohexane
7	7.476	0.3	Undecylcyclohexane
8	7.689	1.31	Undecylcyclohexane
9	7.972	0.68	2-Nitro-tertiary butanol
10	8.509	2.01	*3-n-Propyl-5-methylhexan-2-one*
11	8.755	1.28	3-Isopropyl-5-methylhexan-2-one
12	8.818	1.27	3-Isopropyl-5-methylhexan-2-one
13	9.516	1.74	Dihydro citronellyl angelate
14	10.218	1.64	2-methoxy-3-(2-propenyl)phenol
15	11.324	0.4	trans-Caryophyllene
16	11.815	0.42	α-Humulene
17	12.021	0.81	α-Copaene
18	12.583	0.71	δ-Cadinene
19	12.782	0.5	Selin-6-en-4α-ol
20	13.862	0.55	(1R,3E,7E,11R)-1,5,5,8-Tetramethyl-12-oxabicyclo[9.1.0]dodeca-3,7-diene
21	14.037	1.39	Junenol
22	14.357	1.18	Ar-tumerone
23	15.327	0.66	Phenol, 5-(1,5-dimethyl-4-hexenyl)-2-methyl-, (R)-
24	16.29	0.85	Neophytadiene
25	16.347	0.27	6,10,14-trimethyl-2-Pentadecanone
26	17.24	3.4	Hexadecanoic acid, methyl ester
27	17.607	4.95	n-Hexadecanoic acid
28	18.55	0.8	Kaur-16-en-18-oic acid, methyl ester, (4.beta.)-
29	18.991	2.51	9,12-Octadecadienoic acid (Z,Z)-, methyl ester
30	19.054	2.13	Linolenic acid
31	19.165	7.34	Phytol
32	19.304	0.68	Methyl stearate
33	19.377	0.71	9,12-Octadecadienoic acid (Z,Z)-
34	19.437	0.61	9,12,15-Octadecatrienoic acid, (Z,Z,Z)-
35	20.242	0.77	Phytol, acetate
36	21.227	0.48	Kaurene

37	21.511	2.43	(1R,4aR,4bS,7S,10aR)-1,4a,7-Trimethyl-7-vinyl-1,2,3,4,4a,4b,5,6,7,8,10,10a-dodecahydrophenanthrene-1-carbaldehyde
38	22.06	25.71	Grandiflorenic acid
39	23.732	7.78	Kaurenoic acid
40	24.156	6.8	Nandrolone phenpropionate
41	24.602	0.32	Tetracontane
42	24.837	1.16	Hexadecanoic acid, 2-hydroxy-1-(hydroxymethyl)ethyl ester
43	25.297	1.67	Bis(2-ethylhexyl) phthalate
44	26.053	1.05	(1R,3aS,5aS,8aR)-1,3a,5a-Trimethyl-4-methylenedecahydrocyclopenta[c]pentalene
45	31.772	4.35	Squalene
46	32.175	1.51	9.beta.-Acetoxy-3,5.alpha.,8-trimethyltricyclo[6.3.1.0(1,5)]dodec-3-ene
47	32.264	0.32	Hexadecane
48	33.132	0.52	Neryl linalool isomer

Table S2. GC-MS analysis of *Wedelia triolobata* (L.) chloroform fraction

**Peak number**	**Retention time, min**	**Area, %**	**Compound names**
1	10.35	0.3	5,5-dimethyl-1-propylcyclopenta-1,3-diene
2	13.632	0.25	1-p-Menthen-9-al
3	14.824	0.16	4,6,6-trimethyl-2-(3-methyl-buta-1,3-dienyl)-3-oxa-tricyclo[5.1.0.0 2,4]octane
4	15.122	0.16	4-Ethyl-3-ethylidene-1,1,4-trimethyl-1,2,3,4-tetrahydro-2-naphthalenol
5	15.212	0.12	(E)-4-(3-Hydroxyprop-1-en-1-yl)-2-methoxyphenol
6	15.315	0.15	(-)-Caryophyllene oxide
7	15.434	0.1	Octadecanamide
8	15.522	0.08	Benzoic acid, 4-hydroxy-3,5-dimethoxy-, hydrazide
9	15.651	0.13	2-Acetoxy-1,1,10-trimethyl-6,9-epidioxydecalin
10	16.034	0.18	1-Heptatriacotanol
11	16.387	0.14	Retinal
12	16.527	0.11	Naphthalene-2-carboxylic acid, 3-hydroxy-, (benzo[1,2,5]oxadiazol-5-ylmethylene)hydrazide
13	17.124	0.25	7,9-Di-tert-butyl-1-oxaspiro(4,5)deca-6,9-diene-2,8-dione
14	17.21	0.15	Retinal
15	17.54	0.08	1-((1S,3aR,4R,7S,7aS)-4-Hydroxy-7-isopropyl-4-methyloctahydro-1H-inden-1-yl)ethanone
16	17.654	0.48	Longifolenaldehyde
17	17.759	0.74	Longifolenaldehyde
18	17.833	0.12	1-Heptatriacotanol
19	17.997	0.17	(-)-Globulol
20	18.049	0.05	2-Hydroxy-3-isopropyl-6-methylcyclohex-2-enone

21	18.133	0.04	Testolactone
22	18.197	0.24	Diepicedrene-1-oxide
23	18.283	0.13	1-Heptatriacotanol
24	18.413	0.62	Boronia butenal
25	18.668	0.59	Methyl dihydroisosteviol
26	18.787	0.56	2,5,5,8a-Tetramethyl-4-methylene-6,7,8,8a-tetrahydro-4H,5H-chromen-4a-yl hydroperoxide
27	18.945	0.34	Boronia butenal
28	19.343	2.22	Longifolenaldehyde
29	19.488	0.12	(1RS,5RS,6RS,7RS)-2,2,6,7-tetramethyl-10-oxatricyclo[[5.2.1.0(!,6)]decan-5-ol
30	19.625	5.46	17α,21-Dihydroxypregn-4-en-3,20-dione, 17,21-methylboronate
31	19.81	1.91	1,3-Di(propen-1-yl)adamantane
32	19.895	0.45	Oleamide
33	20.04	4.88	10exo-Methyl-anti(10,11)-tricyclo[4.3.1.1(2,5)]undec-3-en-10endo-ol
34	20.154	4.12	Tricyclo[4.3.1.1<2,5>]undec-3-en-10-ol, 10-methyl-, stereoisomer
35	20.566	1.06	(7R,8R)-cis-anti-cis-Tricyclo[7.3.0.0(2,6)]dodecan-7,8-diol
36	21.051	0.68	3-Isopropyl-tricyclo[4.3.1.1(2,5)]undec-3-en-10-one
37	21.288	0.32	3-Isopropylidene-tricyclo[4.3.1.1(2,5)]undecan-10-one
38	21.676	0.41	(3R,3aR,4aS,5R,9aS)-3,5,8-Trimethyl-3a,4,4a,5,6,7,9,9a-octahydroazuleno[6,5-b]furan-2(3H)-one
39	22.149	1.12	Ethyl linoleate
40	22.244	7.66	Oleamide
41	24.309	2.72	Erucamide
42	25.124	1.22	(2R,3R,4aR,5S,8aS)-2-Hydroxy-4a,5-dimethyl-3-(prop-1-en-2-yl)-2,3,4,4a,5,6-hexahydronaphthalen-1(8aH)-one
43	25.313	0.57	Isooctyl phthalate
44	25.654	1.45	5H-Cyclopropa[3,4]benz[1,2-e]azulen-5-one, 1,1a,1b,4,4a,7a,7b,8,9,9a-decahydro-7b,9,9a-trihydroxy-3-(hydroxymethyl)-1,1,6,8-tet
45	25.827	0.58	1,2,3,4,6,7,8,8a-Octahydronaphthalene-6,7-diol, 5,8a-dimethyl-3-isopropenyl-, cyclic carbonate, trans-
46	26.203	3.46	Alloaromadendrene oxide-(1)
47	26.841	3.6	Chrysanthenyl acetate
48	27.023	0.89	(2R,3R,4aR,5S,8aS)-2-Hydroxy-4a,5-dimethyl-3-(prop-1-en-2-yl)-2,3,4,4a,5,6-hexahydronaphthalen-1(8aH)-one
49	27.355	20.11	(+)-Ledene
50	27.703	0.96	3β-Bromocholest-5-ene
51	27.88	2.22	Alloaromadendrene oxide-(1)
52	29.347	25.37	Linderalactone
